# Risk Factors and Clinical Features of Herpes Zoster in Children: A Case Series

**DOI:** 10.7759/cureus.92147

**Published:** 2025-09-12

**Authors:** Anna Strozak, Elwira Misztela-Lisiecka, Grazyna Waska, Piotr Nowakowski, Magdalena A Miernik-Skrzypczak

**Affiliations:** 1 Internal Medicine, Dr. Anna Gostynska Wolski Hospital, Warsaw, POL; 2 Medicine, Pabianice Medical Centre, Pabianice, POL; 3 Internal Medicine, Specialist Hospital No. 1 in Bytom, Bytom, POL; 4 Internal Medicine, Municipal Hospital in Gliwice, Gliwice, POL; 5 General Medicine, Lower Silesian Center of Oncology, Pulmonology and Hematology, Wrocław, POL

**Keywords:** case report, herpes zoster, immunocompromised, leukemia, pediatric herpes zoster, ramsay hunt syndrome, varicella-zoster virus

## Abstract

Herpes zoster (HZ) is an infectious disease resulting from the reactivation of the varicella-zoster virus (VZV), which remains latent in ganglionic neurons after primary varicella infection. The occurrence of HZ in the pediatric population is relatively uncommon, and identifying risk factors that facilitate VZV reactivation is important. This study presents three cases of children with distinct clinical manifestations of HZ, considering various predisposing factors for disease development. The described patients included a three-year-old boy with a typical form of HZ during maintenance therapy for acute lymphoblastic leukemia (ALL). The second case was a 16-year-old girl with disseminated HZ and a diagnosis of acute myeloid leukemia (AML) who had multiple treatment-related complications. The third patient was an immunocompetent 15-year-old boy with Ramsay Hunt syndrome (RHS). Variables analyzed included immune status, the presence of comorbidities, administered medications, and a history of varicella infection. Based on available literature and case analysis, selected risk factors were discussed, including early childhood varicella infection, acquired immunodeficiencies, and conditions requiring immunosuppressive therapy. This study emphasizes the importance of early identification of at-risk children. Such an approach may facilitate the consideration of preventive strategies and, in the case of symptomatic infection, lead to earlier diagnosis and more effective treatment.

## Introduction

Herpes zoster (HZ) results from the reactivation of latent varicella-zoster virus (VZV) within neurons of cranial nerve ganglia, dorsal root ganglia, and autonomic ganglia [1]. Clinically, it manifests as a polymorphic rash on an erythematous base with a characteristic evolution of lesions. Initially, it is maculopapular and then rapidly progresses to vesicles filled with clear fluid, which subsequently become pustular before crusting [2]. The rash phase is preceded by prodromal symptoms such as pain and skin hyperesthesia within the affected dermatome. It may persist throughout the course of the disease and even for many years afterward, although postherpetic neuralgia occurs less frequently in children than in adults [3,4]. Skin lesions are typically localized within a single dermatome and do not cross the midline of the body. In immunocompromised individuals, HZ may present in a disseminated form and be associated with visceral complications [2]. One distinctive but rare form is Ramsay Hunt syndrome (RHS), or HZ oticus, accompanied by ipsilateral facial nerve palsy. It results from VZV reactivation involving the geniculate ganglion of the facial nerve. Consequently, it presents with symptoms such as a vesicular rash around the auricle, otalgia, facial asymmetry, and taste disturbances. The proximity of the infection to the vestibulocochlear nerve may lead to hearing loss (in approximately 24% of patients), tinnitus, and vertigo [2,5,6]. Beyond its clinical manifestations, understanding the factors that predispose children to HZ is essential for proper evaluation and management. In the pediatric population, immune disorders represent the predominant risk factor for HZ, particularly defects in T-cell-mediated immunity. These include congenital immunodeficiencies and acquired conditions, such as malignancies, infectious diseases, diabetes, and malnutrition, as well as those associated with immunosuppressive therapy. In addition, varicella infection during the first year of life and an incomplete varicella vaccination schedule have been reported as additional predisposing factors [7].

## Case presentation

Case 1

A three-year-old boy had been diagnosed two years earlier with acute lymphoblastic leukemia (ALL) and was undergoing maintenance therapy with mercaptopurine (37.5 mg and 25 mg on alternating days) and methotrexate (13 mg once weekly). He was admitted to the Emergency Department (ER) with a suspicion of HZ. Three days prior to admission, he developed an erythematous, 4 cm lesion with multiple vesicles on its surface, located on the lateral aspect of the left knee. He had a history of varicella in infancy. His medical history was otherwise unremarkable, with normal psychomotor development and no chronic conditions or allergies. Until the diagnosis of leukemia, he had been vaccinated according to the National Immunization Program (NIP) but had not received varicella vaccination, which is not mandatory in Poland.

On admission, the boy was in satisfactory general condition, afebrile, and reported no pain. Vital signs were within normal limits for age. Physical examination revealed no significant abnormalities except for a vesicular rash on the lateral aspect of the left thigh, just above the knee. Laboratory tests showed red blood cell (RBC) count of 3.49 × 10^6^/μL, hemoglobin level of (Hgb) 11.2 g/dL, white blood cell (WBC) count of 9.42 × 10^3^/μL with a predominance of neutrophils (NEU), platelet (PLT) count of 228 × 10^3^/μL, C-reactive protein (CRP) concentration of 0.18 mg/dL (normal value less than 0.5 mg/dL), creatinine level of 0.19 mg/dL, and normal urea and electrolyte levels. Liver transaminase levels were elevated, a finding consistent with the patient’s chronic condition (Table 1). A swab from the vesicular fluid was collected for VZV testing.

**Table 1:** Laboratory findings of Patient 1 on admission * indicates values outside the reference range The peripheral blood smear showed NEU predominance with a reduced proportion of LYMPH compared to the age-appropriate reference range, most likely related to ALL treatment and the concurrent VZV infection. The low creatinine value was considered not clinically significant, most likely reflecting age-related muscle mass rather than impaired renal function. Abbreviations: CBC–Complete Blood Count; NEU–Neutrophils; LYMPH–Lymphocytes; ALT–Alanine aminotransferase; AST–Aspartate aminotransferase; ALL–Acute lymphoblastic leukemia

Laboratory parameter	Patient’s value	Reference range
CBC		
Hgb (g/dL)	11.2	10.9–14.2
Hematocrit (%)	32.7*	34.0–41.0
RBC (×10^6^/µL)	3.49*	4.30–5.50
WBC (×10^3^/µL)	9.42	4.50–13.00
NEU (%)	82.9*	35.0–45.0
LYMPH (%)	8.5*	29.6–69.2
PLT (×10^3^/µL)	228	150–400
Liver function tests		
ALT (U/L)	123*	<36
AST (U/L)	134*	<53
Inflammatory marker		
CRP (mg/dL)	0.18	<0.5
Metabolic parameters		
Glucose (mg/dL)	177	70–200
Creatinine (mg/dL)	0.19*	0.31–0.47
Urea (mg/dL)	32.7	6.4–34.3
Electrolytes		
Potassium (mmol/L)	4.2	3.5–5.1
Sodium (mmol/L)	139	130–143

**Figure 1:**
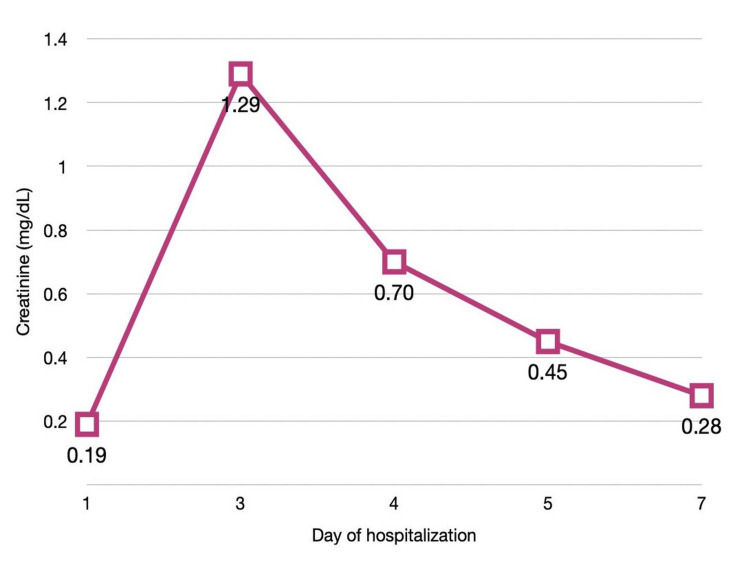
Changes in serum creatinine levels during hospitalization Serum creatinine concentration (mg/dL) measured at admission (day 1) and on selected days during hospitalization An increase was observed on day 3, with subsequent improvement and decline after fluid therapy was initiated.

The patient received intravenous (IV) acyclovir at a dose of 10 mg/kg every eight hours, administered by slow infusion over one hour. Following a hematology consultation, immunosuppressive treatment was temporarily discontinued. During this period, hydration was exclusively oral and was likely suboptimal. On the third day of acyclovir therapy, laboratory tests revealed increased urea (51.3 mg/dL) and creatinine (1.29 mg/dL) levels. Acute kidney injury (AKI) was diagnosed and successfully treated with intensive fluid therapy (Figure 1). On the same day, VZV infection was confirmed by a positive PCR test result obtained from vesicular fluid.

**Figure 2:**
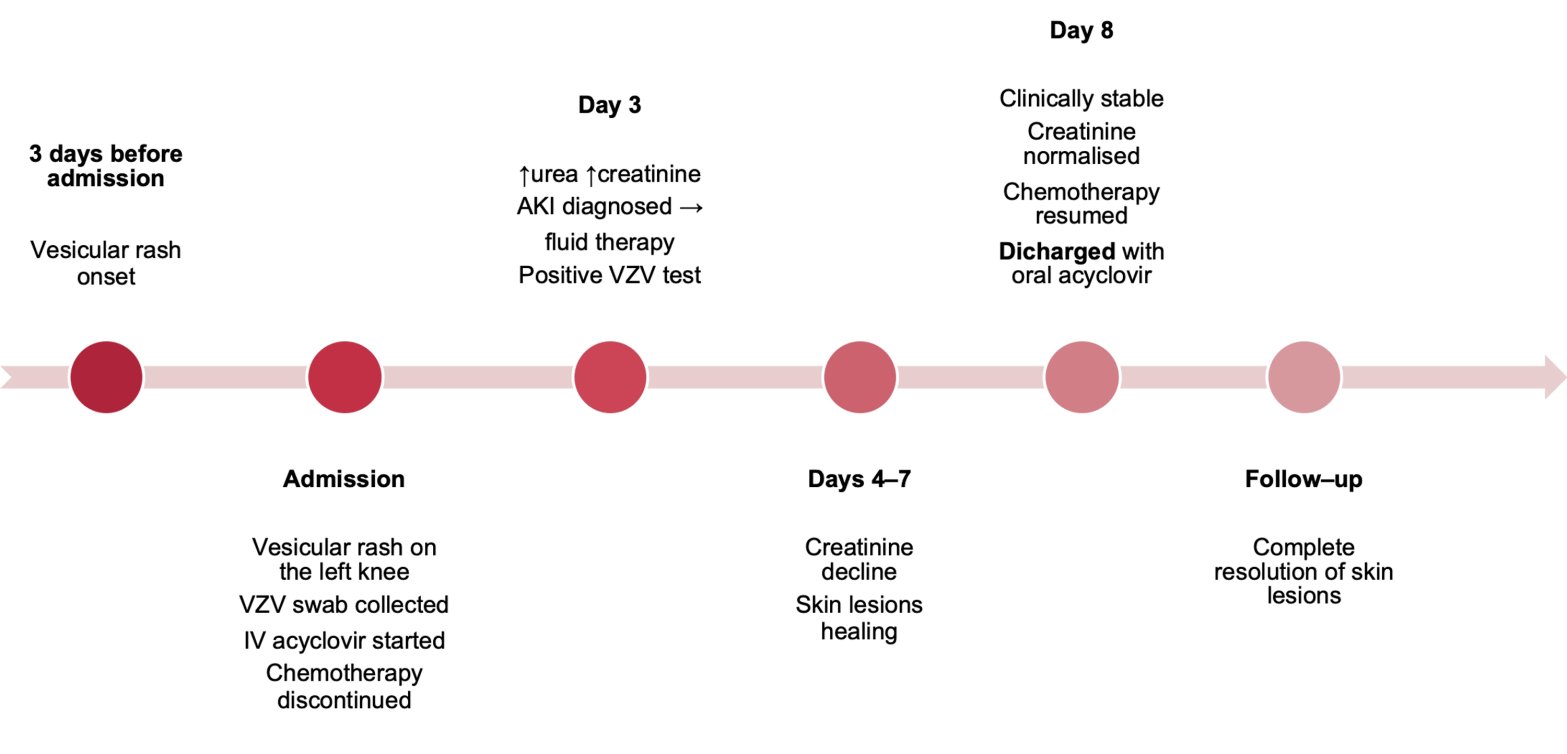
Timeline of Patient 1 Abbreviations: VZV–Varicella-Zoster Virus

Throughout hospitalization, the child remained clinically stable and afebrile. He required no analgesics, and his skin lesions healed appropriately. On the eighth day of treatment, he was discharged home with instructions to continue oral acyclovir at a dose of 20 mg/kg for an additional four days. Mercaptopurine and methotrexate were restarted at the time of discharge. At the follow-up visit, complete resolution of skin lesions was noted. The clinical course of the patient is summarized in the timeline (Figure 2).

Case 2

A 16-year-old girl diagnosed with acute myeloid leukemia (AML) after her third cycle of chemotherapy was admitted to the Hematology Unit for routine follow-up examinations. Her prior treatment was complicated by a pulmonary fungal infection, *Candida* *albicans* sepsis, systemic capillary leak syndrome (SCLS) with pleural effusions, and pericarditis. At the time of hospitalization, she was still receiving voriconazole and prednisone for these conditions.

On admission, the patient was clinically stable considering her underlying disease, with normal vital signs and no fever. A physical examination revealed significant edema and erythema in the right labia majora. During her previous hospitalization, a small pustular lesion had been noted in the same area. The current severity of the inflammatory skin changes was attributed to hematologic reconstitution and an increase in WBC count. Additionally, the patient appeared markedly pale and severely underweight (4th percentile for age and height). No other abnormalities were detected on examination.

Laboratory tests revealed pancytopenia with RBC count of 2.63 × 10^6^/μL, Hgb level of 7.6 g/dL, WBC count of 1.04 × 10^3^/μL, neutrophil (NEU) count of 0.54 × 10^3^/μL, and platelet (PLT) count of 24 × 10^3^/μL. Inflammatory markers were elevated, with a CRP concentration of 1.3 mg/dL and a D-dimer level of 2325 ng/mL (normal value less than 500 ng/mL), while renal function and electrolytes remained within normal limits (Table 2).

**Table 2:** Laboratory findings of Patient 2 on admission * indicates values outside the reference range Abnormalities in CBC and organ function markers may reflect the underlying AML and its treatment. The slightly shortened aPTT was considered to have no major diagnostic significance. Elevated fibrinogen and D-dimer levels may be associated with an inflammatory response. Increased antithrombin activity may also occur in the setting of inflammation or altered liver function. Abbreviations: CBC–Complete Blood Count; NEU–Neutrophils; LYMPH–Lymphocytes; GGT–Gamma-glutamyl transferase; PT–Prothrombin time; aPTT– Activated partial thromboplastin time; ALT–Alanine aminotransferase; AST–Aspartate aminotransferase

Laboratory parameter	Patient’s value	Reference range
CBC		
Hgb (g/dL)	7.6*	12.0–16.0
Hematocrit (%)	21.4*	37.0–47.0
RBC (×10^6^/µL)	2.63*	3.80–5.20
WBC (×10^3^/µL)	1.04*	4.00–10.00
NEU (%)	52	50.0–70.0
Absolute NEU count (x10^3^/µL)	0.54*	1.50–7.50
LYMPH (%)	11.5*	18.0–42.0
Absolute LYMPH count (x10^3^/µL)	0.12*	1.00–4.00
PLT (×10^3^/µL)	24*	150–400
Liver function tests		
ALT(U/L)	57*	<47
AST (U/L)	41	<46
GGT (U/L)	152*	<33
Albumin (g/dL)	3.6	3.2–4.5
Inflammatory marker		
CRP (mg/dL)	1.30*	<0.5
Metabolic parameters		
Glucose (mg/dL)	72	70–200
Creatinine (mg/dL)	0.79	0.50–0.90
Urea (mg/dL)	35.7*	8.6–32.1
Uric acid (mg/dL)	2.7	2.4–6.6
Electrolytes		
Potassium (mmol/L)	3.5	3.5–5.1
Sodium (mmol/L)	137	130–143
Coagulation profile		
PT (s)	12.1	10.10–13.50
aPTT (s)	24.4*	26.0–37.0
Fibrinogen (g/L)	3.73*	1.80–3.50
Antithrombin (%)	133*	75.0–125.0
D-dimer (ng/mL)	2325*	<500

A swab culture from the labial lesion collected during previous hospitalization grew *Klebsiella pneumoniae *producing extended-spectrum β-lactamase (ESBL). The same pathogen was isolated from stool cultures. Based on antibiotic susceptibility testing, treatment with imipenem and cilastatin was initiated. Due to pancytopenia, the patient required transfusions of leukoreduced and irradiated RBC and PLT concentrates, which were well-tolerated without complications.

**Figure 3:**
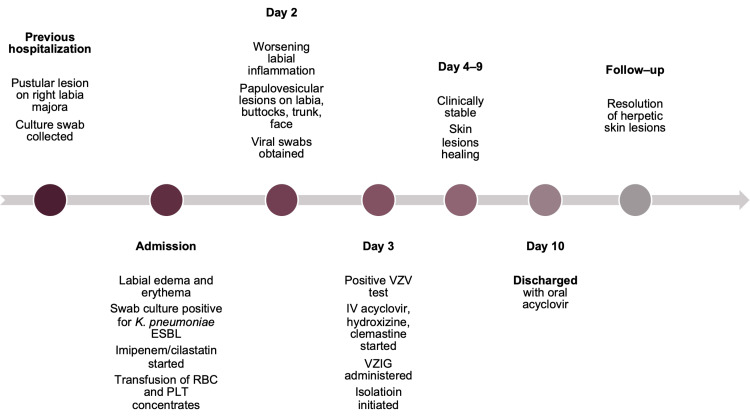
Timeline of Patient 2 Abbreviations: VZV–Varicella-zoster virus; VZIG–Varicella-zoster immunoglobulin; ESBL–Extended-spectrum beta-lactamase

The following day, labial edema progressed, and papulovesicular skin lesions appeared in the area, extending toward the lower abdomen and right buttock. Similar lesions were observed on the trunk, neck, and face. Vesicular fluid from a facial lesion was collected for viral testing, including VZV, herpes simplex virus type 1 and type 2 (HSV-1 and HSV-2), and enterovirus. Based on the clinical presentation, a positive PCR test for VZV, and a history of prior varicella infection, a diagnosis of disseminated HZ was made. The patient received IV acyclovir at a dose of 10 mg/kg every 8 hours, along with continuation of antibiotic therapy and genital area care. Antipruritic treatment with hydroxyzine and clemastine was added. Given the pancytopenia and the generalized presentation of the disease, the patient was administered varicella-zoster immunoglobulin (VZIG) at a dose of 1 mL/kg (45 mL in total, equivalent to 1125 IU) and placed under strict isolation in the Infectious Diseases Unit. In the following days, the patient’s condition remained stable, skin lesions healed appropriately, and labial edema gradually decreased. On the 10th day of acyclovir therapy, she was discharged with instructions to continue oral treatment, 800 mg four times daily for at least 2 weeks. At follow-up, complete resolution of the HZ lesions was confirmed. The clinical course of the patient is summarized in the timeline (Figure 3).

Case 3

A 15-year-old boy with no history of chronic diseases was admitted to the ER due to left-sided facial nerve palsy. Earlier that day, he had been discharged from another hospital where he was diagnosed with HZ oticus. The patient was not receiving any chronic medications and had documented allergies to amoxicillin and cow's milk proteins. He had been vaccinated according to the NIP, but had not received the varicella vaccine. He had a history of varicella at the age of one year.

About 10 days before hospitalization, he developed symptoms of an upper respiratory tract infection, including rhinorrhea and pharyngitis. Two days later, the boy developed pain around the left ear, with edema and erythema of the auricle. Suspecting an external ear infection, topical ciprofloxacin was initiated on an outpatient basis, but no improvement was observed. In the following days, severe pain in the left temporal region, dizziness, and balance disorders developed. The patient was admitted to the hospital, where, based on clinical and otoscopic examination, HZ oticus was diagnosed. After three days of IV acyclovir treatment, he was discharged home with a recommendation to continue oral therapy. That same evening, the patient’s neurological condition worsened. He experienced increased pain and facial asymmetry characterized by drooping of the left corner of the mouth and weaker closure of the left eyelid. Numbness, tingling, and unilateral loss of taste sensation were also noted.

The boy was rehospitalized and consulted by a neurologist and an otolaryngologist. On examination, facial asymmetry was assessed as grade II on the House-Brackmann scale, which corresponds with normal symmetry and muscle tone at rest, as well as complete eye closure with minimal effort [8]. Slight asymmetry of the mouth and flattening of the left nasolabial fold were also noted. No other neurological abnormalities were observed. Hearing was assessed clinically during the physical examination and found to be normal; therefore, audiometry was not considered necessary. Otoscopic examination revealed vesicular lesions and drying crusts on the left auricle and external auditory canal. Laboratory tests revealed no abnormalities (Table 3).

**Table 3:** Laboratory findings of Patient 3 on admission * indicates values outside the reference range A slightly reduced NEU percentage was considered not clinically significant, as all other laboratory values were within the normal range. Abbreviations: CBC–Complete Blood Count; NEU–Neutrophils

Laboratory parameter	Patient’s value	Reference range
CBC		
Hgb (g/dL)	15.5	14.0–18.0
Hematocrit (%)	43.6	40.0–54.0
RBC (×10^6^/µL)	5.15	4.00–6.30
WBC (×10^3^/µL)	7.71	4.00–10.00
Neutrophils (%)	48.1*	50.0–70.0
Lymphocytes (%)	40.9	24.7–56.0
PLT (×10^3^/µL)	347	150–400
Inflammatory marker		
CRP (mg/dL)	<0.060	<0.5
Metabolic parameters		
Glucose (mg/dL)	103	70–200
Creatinine (mg/dL)	0.86	0.70–1.20
Urea (mg/dL)	41.7	10.7–42.8
Electrolytes		
Potassium (mmol/L)	4.4	3.5–5.1
Sodium (mmol/L)	138	130–143

**Figure 4:**
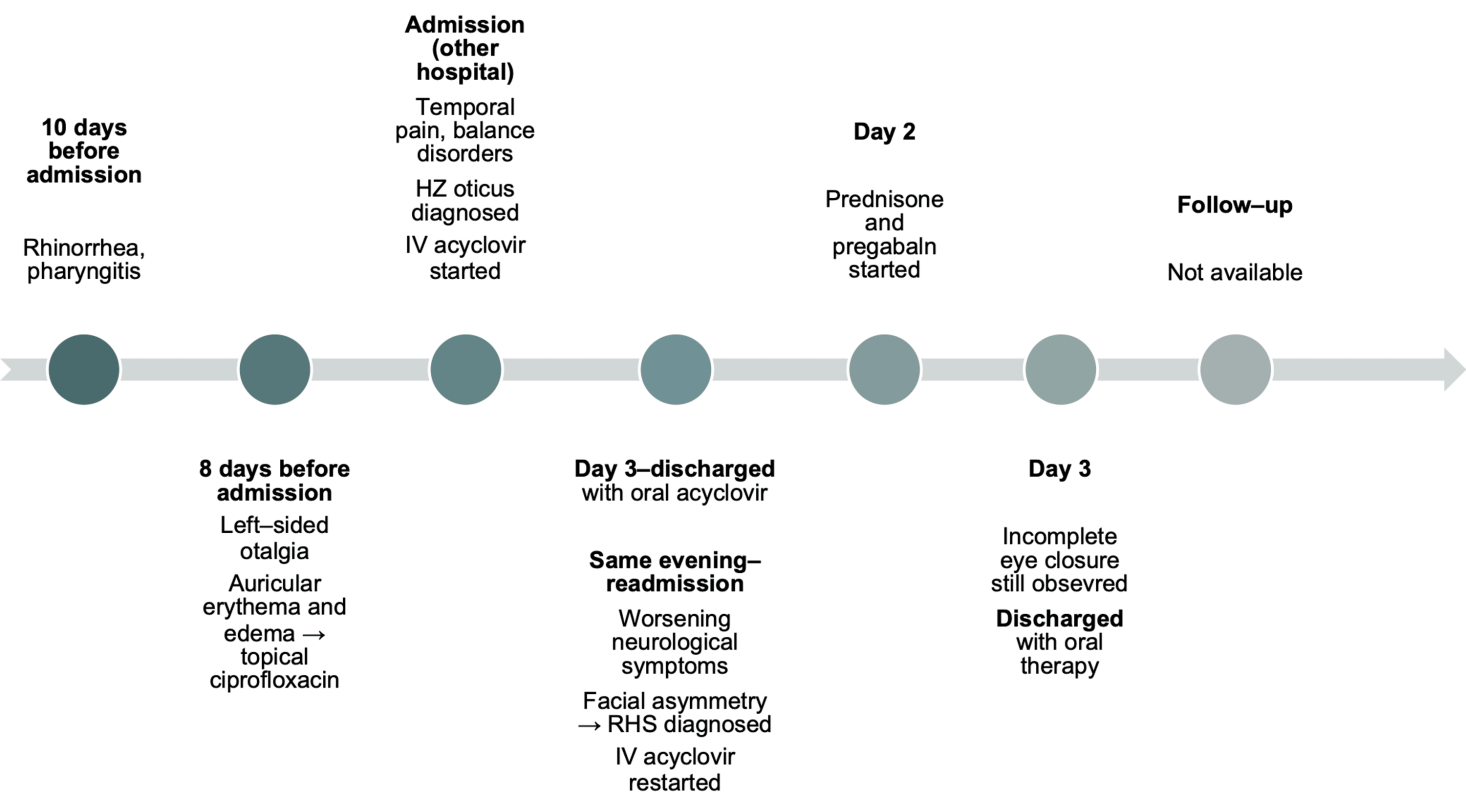
Timeline of Patient 3 RHS–Ramsay Hunt syndrome

Based on peripheral facial nerve paralysis and symptoms of HZ oticus, RHS was diagnosed. The patient received IV acyclovir at a dose of 10 mg/kg every 8 hours, prednisone 30 mg twice daily, and pregabalin 75 mg twice daily. Additionally, eye care with artificial tears and nighttime protective dressings was prescribed to prevent corneal drying. After 3 days of hospitalization, the patient was discharged in stable condition with instructions to continue oral acyclovir 800 mg 4 times daily, prednisone 20 mg twice daily for 5 more days, and neurological rehabilitation. At discharge, a slight, incomplete closure of the left eyelid was still observed. No information about the patient’s subsequent condition or clinical outcome was available due to a lack of follow-up. The clinical course of the patient is summarized in the timeline (Figure 4).

Informed consent for publication was obtained from the patients’ legal guardians, and assent was obtained from the two older children.

## Discussion

In this series of three pediatric HZ cases, we present a patient with ALL and a typical course of HZ, a patient with AML and disseminated HZ, and a patient with RHS. The cases illustrate the varied clinical presentations of HZ in children, which differ according to immune status, comorbidities, and other predisposing factors for disease development (Table 4).

**Table 4:** Comparative summary of clinical features, risk factors, and treatment outcomes in three pediatric patients diagnosed with HZ Abbreviations: HZ–Herpes zoster

Parameter	Case 1	Case 2	Case 3
Age/sex	3 M	16 F	15 M
Comorbidities	ALL	AML	None
Immunosuppression	Yes	Yes	No
Risk factors	Malignancy, immunosuppression, infantile varicella	Malignancy, immunosuppression	Viral infection, varicella at one year of age
Form of HZ	Limited to one dermatome	Disseminated HZ	RHS
Location of skin lesions	Left knee region	Right labia majora, buttocks, trunk, face	Left auricle, external auditory canal
Other symptoms/organ involvement	AKI	Pain, pruritus	Headache, left-sided otalgia, left-sided facial nerve palsy, vertigo
Treatment	Acyclovir	Acyclovir, VZIG, hydroxyzine, clemastine	Acyclovir, prednisone, pregabalin
Hospitalization duration	8 days	10 days	3 days
Outcome	Full recovery	Full recovery	Not available

Compared to adults, HZ in children occurs relatively rarely, which limits the availability of epidemiological studies in this age group. According to Weinmann et al., in a population-based study conducted on over 6 million children in the United States, the incidence was 74 per 100,000 person-years. The risk of developing HZ increased with age, reaching the highest incidence in the 10-17-year-old age group. Additionally, the study indicated that children vaccinated against varicella had a significantly lower incidence of HZ than unvaccinated children (38 vs. 170 per 100,000 person-years) [9]. Simpson et al., based on a literature review, demonstrated that in countries with routine varicella vaccination programs, the incidence of HZ is lower compared to countries without such programs [10]. Cases of RHS have also been described in children, although they are rare, with an incidence of approximately 2.7 per 100,000 person-years. RHS accounts for approximately 10% of peripheral facial nerve palsy cases in the pediatric population and is the second most common cause of non-traumatic facial nerve palsy [6].

Pathophysiologically, VZV reactivation results from impaired cellular immunity, primarily involving CD4+ and CD8+ T lymphocytes. In chronic infections caused by *Herpesviridae*, CD4+ T-cells support the functions of CD8+ T-cells and B-cells and exert direct effects by secreting cytokines such as interferon-gamma (IFN-γ), which possesses potent antiviral properties. A deficiency in these lymphocytes leads to increased viral replication and reactivation of infection [11]. This pattern is observed in conditions such as primary immunodeficiencies, hematopoietic malignancies and other neoplasms, immunosuppressive therapy, hematopoietic stem cell transplantation (HSCT), solid organ transplantation, and human immunodeficiency virus (HIV) infection [7].

According to published data, immunocompromised children have a five to six-fold higher incidence of HZ than immunocompetent children [9]. Gadimova et al. analyzed 150 pediatric HZ cases and found that 79.3% occurred in children with comorbidities that impair immunity, mainly hematologic and solid tumor types, as well as in those post-HSCT. The largest subgroup consisted of children with ALL. Immunosuppression was associated with longer hospitalization (median 7 vs. 5 days in healthy children) and a significantly higher rate of hepatitis (20% vs. 0%). Disseminated and recurrent forms of HZ were also reported in this group [12]. Lin et al. demonstrated an increased risk of HZ in children with malignancies, with leukemia cases showing the strongest association [13].

Children with ALL and AML are particularly susceptible to reactivation of latent VZV due to bone marrow infiltration by malignant cells, which displace hematopoietic stem cells (HSCs) from their normal niches. The uncontrolled proliferation of blasts impairs hematopoiesis by occupying the bone marrow niche and disrupting the microenvironment that normally supports HSCs’ survival, proliferation, and differentiation [14]. Additionally, chemotherapy used in treating these malignancies exerts cytotoxic effects on rapidly dividing cells, including those of the immune system, leading to lymphopenia and impaired immune control of latent VZV. Sørensen et al. analyzed 226 pediatric ALL cases and found that HZ developed in 29% of the children during primary chemotherapy. A higher incidence was observed in patients treated under high-risk protocols, correlating with treatment intensity [15].

The patient with ALL described in our study received immunosuppressive treatment with mercaptopurine and methotrexate, which predisposes to VZV reactivation. Despite this, he presented with a classic dermatomal course, without systemic symptoms or complications. Antiviral therapy was effective; however, the risk of acute kidney injury and the need for adequate hydration in patients treated with acyclovir should be considered [16]. In contrast, the patient with AML developed disseminated HZ, a particularly atypical presentation, indicative of profound cellular immune impairment during chemotherapy. In addition to antiviral therapy with acyclovir, specific VZIG was administered, consistent with current recommendations for treating HZ in immunosuppressed patients with severe disease [17].

The case of an immunocompetent child who developed RHS at age 15 was atypical because the patient had no known immune disorders or chronic diseases. Therefore, other risk factors that may contribute to pediatric HZ should be considered. Acute viral infections may transiently weaken cellular immunity. Although there are few studies directly associating upper respiratory viral infections with HZ occurrences, it is known that such infections can lead to a temporary impairment of immune system function [10]. Additionally, it is worth noting that two of the patients had a history of varicella at approximately one year of age. Data suggest that immune immaturity at this age may lead to a suboptimal response to primary varicella infection, increasing the risk of early VZV reactivation and HZ at a young age [4].

In all the presented clinical cases, early initiation of antiviral therapy with acyclovir resulted in clinical improvement and prevented the development of complications. Initiating acyclovir treatment within 72 hours of symptom onset has been shown to reduce the formation of new vesicles and accelerate the healing of skin lesions [2,18]. Additional important measures include patient isolation to reduce the risk of transmission to other immunocompromised children, adequate hydration during acyclovir therapy to minimize nephrotoxicity, and temporary discontinuation of immunosuppressive treatment during the active phase of infection. These cases highlight the importance of clinical vigilance and rapid diagnosis, particularly in atypical disease presentations.

A limitation of this study is the small number of cases; nevertheless, the analysis provides valuable practical insights and emphasizes the importance of early identification of at-risk children and prompt therapeutic intervention in this population.

## Conclusions

HZ in children most commonly occurs in the context of immunocompromised states but may also affect otherwise healthy children. Our cases illustrate that HZ may present in children with malignancy or undergoing immunosuppressive treatment. Varicella in early childhood and other viral infections may predispose children to VZV reactivation. The presented cases highlight the importance of clinical vigilance in children with identified risk factors. Early diagnosis, prompt antiviral therapy, and consideration of preventive strategies are crucial for minimizing the risk of severe cases and complications in this population.
